# Transformative Outcomes With Paliperidone Long-Acting Injection in Severe Treatment-Resistant Schizophrenia: A Case Report and Literature Review

**DOI:** 10.7759/cureus.65939

**Published:** 2024-08-01

**Authors:** Syed Ali Bokhari, Daliya Al Maallah, Ghina Alramahi, Alma Al Mansour, Abdelaziz Osman

**Affiliations:** 1 Psychiatry, Al Amal Psychiatric Hospital, Emirates Health Services, Dubai, ARE; 2 Emergency Medicine, Al Qassimi Hospital, Emirates Health Services, Sharjah, ARE

**Keywords:** very-early-onset schizophrenia (veos), paliperidone palmitate, paliperidone lai, treatment-resistant, atypical antipsychotic, treatment-resistant schizophrenia, paliperidone, schizophrenia

## Abstract

Treatment-resistant schizophrenia (TRS) presents considerable challenges in contemporary psychiatric practice due to inadequate response to conventional antipsychotic treatments. Paliperidone, the primary active metabolite of risperidone, particularly in its long-acting injectable (LAI) form, has emerged as a promising option for TRS due to its consistent medication delivery, reducing symptom exacerbation and relapse associated with oral dosing fluctuations. This case report presents the clinical journey of a 42-year-old female diagnosed with schizophrenia at age 15. Despite numerous hospital admissions and trials of various oral and injectable antipsychotics, including clozapine and electroconvulsive therapy (ECT), her symptoms persisted. During her last admission, her condition showed minimal improvement despite extensive pharmacological interventions. Introducing paliperidone LAI while tapering off other antipsychotics led to significant improvements within four weeks. The patient exhibited reduced hallucinatory behaviour, delusions, and disorganized behaviour. Follow-up assessments confirmed sustained progress, with the patient showing increased engagement in daily activities and reduced irritability and suspiciousness. This case underscores the potential efficacy of paliperidone LAI in managing TRS. The patient's notable improvement highlights the importance of personalized treatment plans and continuous monitoring in complex psychiatric conditions. Its favourable safety and tolerability profile further supports its use as a long-term treatment option for TRS, potentially leading to enhanced patient compliance and overall quality of life. The significant symptomatic relief and functional improvement observed advocate for the consideration of paliperidone LAI as a promising therapeutic option for TRS, with the potential to be considered in the future among the first-line treatments for TRS.

## Introduction

Schizophrenia is a chronic mental health condition that most often manifests in early adulthood and can lead to episodic and varying levels of disability. DSM-5-TR [1] criteria define schizophrenia as the presence of two or more of five core symptoms (delusions, hallucinations, disorganized speech, grossly disorganized or catatonic behaviour, and negative symptoms). At least one of the symptoms must be delusions, hallucinations, or disorganized speech, and symptoms must be present for at least six months.

Treatment-resistant schizophrenia (TRS) refers to the significant proportion of schizophrenia patients who continue to have symptoms and poor outcomes despite treatment. While there are several definitions of TRS, the majority include failure of two different antipsychotics as a minimum criterion, one usually an atypical, of adequate dose and duration [2-4]. The adequate dose is often considered 400-600 mg chlorpromazine equivalent, and duration varies but is frequently agreed as a minimum of six weeks of treatment [5,6].

The literature reports that up to 30-34% of patients diagnosed with schizophrenia may later develop TRS [7]. TRS poses significant challenges in psychiatric practice, as traditional antipsychotic medications often fail to yield satisfactory results. This necessitates the exploration of alternative therapies to manage the complex symptoms associated with TRS [8].

Antipsychotics are the mainstay of the treatment of schizophrenia. They are classified into first-generation and second-generation antipsychotics (FGA/SGA).

Paliperidone, the primary active metabolite of risperidone, an SGA, especially in its long-acting injectable (LAI) form, has emerged as a promising option for TRS. Its pharmacokinetic profile ensures consistent medication delivery, reducing symptom exacerbation and relapse associated with oral dosing fluctuations. Research indicates that paliperidone LAI may improve both positive and negative symptoms of schizophrenia, decrease relapse risk, and enhance patient compliance and quality of life [9].

This case report presents the clinical journey of a 42-year-old female diagnosed with schizophrenia at age 15. Despite numerous hospital admissions and trials of various oral and injectable antipsychotics, her symptoms, such as self-muttering, a fixed persecutory delusion, auditory and visual hallucinations, disorganized behaviour, and self-neglect, persisted. Extensive therapeutic interventions, including clozapine and electroconvulsive therapy (ECT), showed limited success.

After transitioning to a treatment regimen that included paliperidone LAI, significant improvements in her symptoms and functioning were observed. This case highlights her clinical progression and the efficacy of paliperidone LAI in managing TRS, providing valuable insights into its potential as a viable treatment option.

## Case presentation

A 42-year-old Middle Eastern female, diagnosed with schizophrenia at age 15, has a history marked by multiple admissions to various psychiatric facilities over three decades. Initially presenting in 1997, she was diagnosed with schizophrenia and began regular psychiatric follow-ups by age 20. Despite extensive trials of psychotropic medications including aripiprazole, escitalopram, lorazepam, quetiapine, lamotrigine, haloperidol, risperidone, and flupentixol, her symptoms showed limited-to-no response. She had two partial trials of clozapine, which were discontinued due to poor compliance and adverse effects.

The patient's developmental history was unremarkable, apart from bedwetting until age 10. She began school at age six but left early due to poor performance. Medically, she has a history of Graves' disease treated in 2003, resulting in hypothyroidism post-radioactive iodine therapy in 2007, managed with thyroxine. She had a parathyroid tumour removal in 2023. There is no family history of mental illness, although there is a family history of Down syndrome.

During her most recent admission, the patient exhibited severe psychotic symptoms, including self-muttering, a fixed delusion of persecution, auditory and visual hallucinations, disorganized behaviour, and self-neglect. She believed a recording device was implanted in her throat, through which she was being monitored and plotted against. The auditory hallucinations were commanding and commenting in nature, causing significant distress and leading her to insert tissues into her ears, nostrils, and mouth to quieten the voices. Her behaviour included wandering, aggression, overeating, excessive water intake, and urinary incontinence.

Despite concurrent use of multiple antipsychotics, her symptoms persisted. ECT was attempted, with 12 initial sessions followed by a maintenance course of 20 sessions, but showed limited effectiveness. Following ECT, she continued to exhibit hallucinations, delusions, irritability, and disorganized behaviour.

Upon our team's intervention in July 2022, a revised treatment regimen was implemented due to her history of treatment resistance and polypharmacy. Aripiprazole and trifluoperazine were tapered, risperidone (oral and long-acting injection) was discontinued, and paliperidone long-acting injection (150 mg monthly) was initiated. Once aripiprazole and trifluoperazine were stopped, risperidone oral was reintroduced and gradually titrated upwards alongside the monthly paliperidone long-acting injection. Significant improvements were observed within four weeks. Although she had poverty of thoughts, delayed speech, and psychomotor retardation due to the chronic nature of the illness, the patient no longer exhibited hallucinatory behaviour, delusions of thought control, or aggression, and her disorganized behaviour subsided.

The patient was discharged two months after admission and maintained on risperidone oral (4 mg once daily) and paliperidone long-acting injection (150 mg monthly). Subsequent follow-ups showed sustained progress. The patient, now more interactive and involved in home activities, reported reduced irritability and suspiciousness. Although residual auditory hallucinations and delusions persisted, they were less distressing, and she did not act on them. Her treatment compliance was high, with manageable side effects such as bilateral fine tremors and cogwheel rigidity, managed with procyclidine.

Table 1 depicts the psychotropic medications the patient received each year during her hospital course.

**Table 1:** Psychotropic medications the patient received each year during her hospital course. qAM (quaque ante meridiem): every morning; qPM (quaque post meridiem): every afternoon or evening; q8hr: every eight hours; q12hr: every 12 hours; q24hr: every 24 hours

Year	Psychotropic medications
2011	Sodium valproate 500 mg q12hr
	Aripiprazole 15 mg q24hr
	Clonazepam 0.5 mg q24hr
2012	Sodium valproate 500 mg q12hr
	Aripiprazole 15 mg q24hr
	Clonazepam 0.5 mg q24hr
	Olanzapine 10 mg q24hr
2013	Sodium valproate 500 mg q12hr
	Aripiprazole 15 mg q24hr
	Clonazepam 0.5 mg q24hr
	Olanzapine 10 mg q24hr
2014	Aripiprazole 10 mg q24hr
	Haloperidol long-acting injection, 100 mg, IM, every four weeks
	Escitalopram 10 mg q24hr
	Procyclidine 5 mg q24hr
	Sertindole 20 mg q24hr
	Promethazine 10 mg qPM
2015	Aripiprazole 10 mg q24hr
	Haloperidol long-acting injection, 100 mg, IM, every four weeks
	Escitalopram 10 mg q24hr
	Procyclidine 5 mg q24hr
	Sertindole 20 mg q24hr
	Promethazine 10 mg qPM
	Quetiapine 200 mg qPM
2016	Aripiprazole 10 mg q24hr
	Haloperidol long-acting injection, 100 mg, IM, every four weeks
	Escitalopram 10 mg q24hr
	Procyclidine 5 mg q24hr
	Sertindole 20 mg q24hr
	Promethazine 10 mg qPM
	Quetiapine 200 mg qPM
	Lorazepam 2 mg q24hr
2017	Aripiprazole 10 mg q24hr
	Haloperidol long-acting injection, 100 mg, IM, every four weeks
	Escitalopram 10 mg q24hr
	Procyclidine 5 mg q24hr
	Sertindole 20 mg q24hr
	Promethazine 10 mg qPM
	Quetiapine 200 mg qPM
	Lorazepam 2 mg q24hr
2018	Aripiprazole 10 mg q24hr
	Haloperidol long-acting injection, 100 mg, IM, every four weeks
	Escitalopram 10 mg q24hr
	Sertindole 20 mg q24hr
	Lorazepam 2 mg q24hr
	Trifluoperazine 5 mg q24hr
	Haloperidol 10 mg q12hr
	Benzhexol 5 mg q12hr
	Quetiapine 200 mg q12hr
	Procyclidine 5 mg q12hr
	Flupenthixol long-acting injection, 40 mg, IM, every four weeks
	Promethazine 25 mg qPM
2019	Flupenthixol long-acting injection, 100 mg, IM, every four weeks
	Lorazepam 2 mg q12hr
	Olanzapine 5 mg q12hr
	Quetiapine 400 mg q12hr
	Aripiprazole 30 mg q24hr
	Procyclidine 5 mg q12hr
	Ziprasidone 80 mg q12hr
	Clonazepam 2 mg q24hr
	Promethazine 25 mg qPM
	Haloperidol 5 mg q12hr
2020	Flupenthixol long-acting injection, 100 mg, IM, every four weeks
	Ziprasidone 80 mg q12hr
	Lorazepam 1 mg q24hr
	Promethazine 25 mg qPM
	Haloperidol 5 mg q12hr
	Procyclidine 5 mg q24hr
	Clozapine 100 mg qAM+250 mg qPM
	Paliperidone long-acting injection, 150 mg, IM, every four weeks
	Benzhexol 2 mg q24hr
	Clonazepam 2 mg q12hr
	Risperidone 6 mg q24hr
	Diazepam 5 mg q24hr
2021	Risperidone 1 mg q12hr
	Benzhexol 5 mg q12hr
	Paliperidone long-acting injection, 100 mg, IM, every four weeks
	Quetiapine 200 mg qPM
	Clozapine 100 mg qAM+50 mg qPM
	Diazepam 5 mg q24hr
	Aripiprazole 15 mg q24hr
	Procyclidine 5 mg q24hr
	Risperidone long-acting injection, 50 mg, IM, every two weeks
	Trifluoperazine 5 mg q12hr
2022	Risperidone 1 mg q12hr
	Benzhexol 5 mg q12hr
	Quetiapine 200 mg qPM
	Diazepam 5 mg q24hr
	Aripiprazole 15 mg q24hr
	Procyclidine 5 mg q24hr
	Risperidone 4 mg q12hr
	Paliperidone long-acting injection, 150 mg, IM, every four weeks
2023	Paliperidone long-acting injection, 150 mg, IM, every four weeks
	Risperidone 4 mg q24hr
	Promethazine 50 mg q12hr
	Procyclidine 5 mg q12hr
	Lorazepam 1 mg q12hr

## Discussion

The management of TRS presents a significant challenge in psychiatric practice, as traditional oral antipsychotics often fail to achieve satisfactory outcomes for many patients. However, recent advancements in psychopharmacology, particularly the introduction of paliperidone in its LAI formulation, have offered a promising alternative in treating TRS. A growing body of evidence supports this due to its unique pharmacokinetic profile, efficacy, safety, and tolerability [6,10].

Paliperidone, the primary active metabolite of risperidone, boasts an extended half-life, which is further prolonged in its LAI formulation. The paliperidone palmitate once-monthly formulation (PP1M) is a long-acting ester of paliperidone available as an aqueous nanoparticle suspension for intramuscular injection. Upon administration, it dissolves slowly due to its near-insolubility in water and is hydrolyzed to release paliperidone, the active antipsychotic agent. Paliperidone primarily exerts its effects by antagonizing dopamine D2 and serotonin 5-HT2A receptors, which helps normalize dopamine and serotonin levels implicated in schizophrenia. Additionally, it antagonizes α1 and α2 adrenergic receptors, which are involved in regulating vascular tone and stress responses, and H1 histaminergic receptors, which play a role in wakefulness and appetite regulation. The lack of affinity for M1 cholinergic and β adrenergic receptors reduces side effects such as sedation and cardiovascular impacts. Meanwhile, the paliperidone palmitate once every three months (PP3M) formulation, designed for quarterly administration, uses larger particles compared to PP1M, which slows the release of paliperidone, allowing for extended dosing intervals. Similar to the monthly formulation, PP3M is hydrolyzed to release paliperidone, which acts by antagonizing dopamine D2 and serotonin 5-HT2A receptors, modulating neurotransmitter activity critical in treating psychosis. It also antagonizes α1 and α2 adrenergic and H1 histaminergic receptors, influencing various physiological functions such as blood pressure regulation, stress response, and histamine-related activities. Paliperidone's distinct receptor binding profile compared to risperidone leads to different molecular signalling pathways and therapeutic effects, impacting serotonergic and noradrenergic signalling. These properties make paliperidone an effective antipsychotic with a reduced side effect profile relative to older antipsychotics [11,12].

The extended half-life of paliperidone ensures stable plasma concentrations, reducing the likelihood of non-adherence, a common issue in schizophrenia management. The pharmacokinetic advantages of paliperidone LAI are critical to its effectiveness. It has a sustained-release mechanism and extended half-life that ensure stable plasma concentrations, minimizing the peaks and troughs associated with oral antipsychotics, which can lead to symptom exacerbation and non-adherence. Furthermore, studies have shown that 59% of a single dose of monthly paliperidone LAI (PP1M) is excreted in the urine unchanged, leading to a low potential for drug-drug interactions [2].

Emerging evidence suggests that paliperidone palmitate is an effective treatment option for TRS. TRS patients receiving paliperidone LAI experienced significant improvements in both positive and negative symptoms compared to those on oral antipsychotics. The sustained-release mechanism of the LAI formulation ensures consistent medication delivery, minimizing the peaks and troughs associated with oral dosing, which can exacerbate symptoms and lead to relapse, significantly reducing admission incidence and emergency room visits [13]. Furthermore, it has been indicated that LAI antipsychotics, including paliperidone palmitate, significantly reduce the risk of relapse and hospitalization in TRS patients compared to their oral counterparts, highlighting the importance of this therapeutic approach in mitigating functional decline [14-16].

The economic impact of paliperidone LAI should not be overlooked. Due to the reduced hospitalization rates and overall healthcare costs, it is a cost-effective option for managing TRS, which translates to lower healthcare resource utilization and associated costs. Reduced need for acute psychiatric interventions and better adherence rates also contribute to better long-term management of TRS while maintaining a low potential for adverse effects and drug-drug interactions [17-19].

Safety and tolerability are paramount in the long-term treatment of TRS. Paliperidone has a favourable safety profile that supports its use as a long-term treatment option. Recent pharmacodynamic studies have emphasized the contribution of paliperidone's steady-state levels to its efficacy and tolerability, thereby minimizing adverse effects such as extrapyramidal symptoms and weight gain commonly associated with FGA [20]. Its gradual absorption and prolonged action alleviate the daily burden of side effects, enhancing patient compliance and overall quality of life [21].

Quality of life and functional outcomes are critical in TRS management. Effective symptom control with paliperidone LAI has been associated with better social and occupational functioning, significantly enhancing the overall quality of life for patients. These improvements in daily functioning and social interactions are vital for the long-term well-being of TRS patients [22].

Moreover, recent findings suggest potential cognitive benefits associated with paliperidone LAI, with studies indicating improvements in cognitive function, particularly in memory and executive function domains, compared to oral antipsychotics [23]. These cognitive benefits further support the holistic impact of paliperidone LAI on patient outcomes, addressing both symptomatic and functional aspects of TRS.

Future research directions are also promising. Ongoing research efforts aim to further enhance the treatment landscape for TRS, offering hope for continued improvements in patient outcomes. The three-monthly (PP3M) and six-monthly (PP6M) formulations of paliperidone have been developed to further improve patient outcomes by reducing the frequency of injections, thereby enhancing adherence and overall quality of life. A comparative study highlighted the superior efficacy of paliperidone LAI (both PP1M and PP3M) over other antipsychotic treatments, both oral and injectable (haloperidol decanoate, fluphenazine decanoate, and bromperidol) [23] antipsychotics, in reducing the severity of both positive and negative symptoms in schizophrenia, especially in patients with prior failed treatment of oral antipsychotics or other LAIs, in patients with a history of medication noncompliance, or in patients with an individual preference for less frequent dosing [9]. This reinforces the utility of paliperidone LAI as a viable option for TRS and perhaps can be considered to be among the first-line treatments.

Studies have demonstrated that these extended-interval formulations maintain therapeutic plasma levels effectively and offer similar efficacy in symptom control compared to monthly injections. A study showed that the longer-acting PP6M (in a clinical trial setting) substantially delayed relapse occurrence, with fewer patients experiencing relapse (3.9%) compared with the shorter-acting PP3M (20.2%) and PP1M (29.8%) in routine treatment settings among adults with schizophrenia [24].

The current literature increasingly supports the use of paliperidone LAI as monotherapy for TRS. A large cohort study comprising 29,823 patients compared the relapse prevention rates among different antipsychotics (namely, paliperidone, levomepromazine, perphenazine, zuclopenthixol, olanzapine, risperidone, aripiprazole, clozapine, and quetiapine). Paliperidone LAI was among those with the lowest rates of rehospitalization [25]. Its efficacy in symptom control, favourable pharmacokinetic profile, safety, tolerability, and real-world effectiveness position it as a compelling option in the management of this challenging condition. Further research is warranted to explore its long-term benefits and potential in combination therapies. However, the existing evidence suggests that paliperidone LAI holds promise as a cornerstone in the treatment of TRS, with the potential to be considered in the future among the first-line treatments for TRS.

This case also emphasizes the importance of adherence to treatment regimens and the role of family support in managing chronic psychiatric conditions, through communication, open knowledge exchange, and collaboration. The patient's improvement after transitioning to paliperidone LAI and the discontinuation of ineffective medications suggest that individualized treatment plans are crucial in achieving better outcomes for TRS patients.

## Conclusions

This case report highlights the therapeutic efficacy of paliperidone LAI in managing severe TRS. The patient demonstrated substantial clinical improvement after transitioning to paliperidone LAI, following the limited success of prior antipsychotic treatments, underscoring the necessity for individualized treatment strategies. Paliperidone LAI markedly reduced psychotic symptoms within four weeks, with sustained enhancement of daily functioning and social interactions over time. The decreased frequency of administration facilitated improved compliance, a critical factor in the management of TRS. The stable plasma concentrations provided by paliperidone LAI minimize symptom exacerbation and relapse rates and enhance the overall quality of life of patients, highlighting the potential for it to be considered as a first-line treatment of TRS in the future. It also signifies the importance of tailored treatment plans and continuous monitoring for optimal outcomes in TRS patients.
